# Society Meetings

**Published:** 1913-01

**Authors:** 


### SOCIETY MEETINGS.

Secretaries of Societies in Western N. Y. are requested to send brief notices of meetings. Full transactions will be printed at cost of composition.

---

The Medical Society of the County of Chautauqua held its annual meeting, Dec. 10, at Jamestown. The program included an address by the President, Dr. H. A. Eastman of Jamestown; a paper on Eclampsia by Dr. Garnet L. Hunter of Westfield; one on Treatment of High Blood-pressure by Dr. Fred C. Rice of Ripley; and one upon Duodenal Ulcer by Dr. Charles E. Goodell of Jamestown.

The following officers were elected: President, Dr. N. G. Richmond, Fredonia; vice-presidents, Dr. George F. Smith, Falconer; Dr. F. C. Rice, Ripley; secretary and treasurer, Dr. J. W. Morris, Jamestown.

---

Oneida County Medical Society. In the matter of the proposed action of the Oneida County Medical Society in voting to disclipline Drs. W. B. Reid, J. O. Stranahan, G. C. Reid and J. E. Groff, of Rome, N. Y., an injunction on the Society was served at the July meeting, postponing further action. The interest of the Oneida County Medical Society in this matter have been placed in the hands of Hon. E. M. Willis.

---

At the Annual Meeting of the Elmira Academy of Medicine, Dec. 4, the following officers for 1913 were elected: Pres., A. Mark; vice-president, John A. Bennett; secretary, Chas. Haas; treasurer, C. G. Jennings; trustees, John A. Fisher and A. W. Booth. Program: Report of the Congress of Surgical Clinics, H. W. Fudge; Asexualization, F. L. Christian.

---

The 91st annual meeting of the Medical Society of the County of Erie was held on December 16th, 1912.

Officers for the year 1913, were elected as follows: Dr. J. F. Whitwell, President; Dr. John V. Woodruff, 1st Vice-President; Dr. Arthur W. Hurd, 2nd Vice-President; Dr. Franklin C. Gram, Secretary; Dr. Albert T. Lytle, Treasurer; Dr. John D. Bonnar, Chairman, Board of Censors; Dr. F. Park Lewis, Chairman, Committee on Legislation; Dr. Henry R. Hopkins, Chairman, Committee on Public Health; Dr. Harry Mead, Chairman, Committee on Membership; Drs. Thomas H. McKee, William H. Thornton, Charles A. Wall, George F. Cott and Edward L. Frost,

Delegates to the Medical Society of the State of New York, for the year 1913-1914.

Dr. Fred T. Koyle was elected to membership. Annual reports were submitted by the Treasurer, Chairman of Committee on Public Health and Chairman of the Board of Censors.

---

THE MONROE COUNTY MEDICAL SOCIETY held its annual meeting on December 17th. Considerable business was transacted relative to preparation for the meetings of the New York State Society which are to be held in Rochester April 30 and May 1. The retiring president, Dr. S. W. Little, made an admirable address. The result of the election of officers: President, C. R. Witherspoon; Vice-President, A. C. Snell; Secretary, C. W. Hennington; Treasurer, F. W. Seymour.

---

The HOSPITAL MEDICAL SOCIETY of Rochester, held its annual meeting on Dec. 19. Dr. Wynn B. Palmer read a paper upon the Therapeutic Value of Roentgen Rays. The new officers are: President, W. H. Sutherland; Vice-President, G. H. Gage; Secretary and Treasurer, G. M. Gelsir.

---

THE ROCHESTER PATHOLOGICAL SOCIETY has held meetings as follows: Nov. 29, Dr. C. L. Hericher read a paper on Gastric and Duodenal Ulcers. Dec. 12, Dr. A. M. Loewenstein on The Social Evils. Dec. 27, Dr. F. C. Goldsborough of Buffalo, on Toxaemias of Pregnancy.

---

THE ROCHESTER ACADEMY OF SCIENCE, on Dec. 9th, listened to an address by Dr. A. L. Benedict on Food Values, Physiologic and Oeconomic.

---

THE BUFFALO MEDICAL AND SURGICAL LEAGUE held its December meeting at the Statler Hotel. Dr. Benjamin Jacobson read a paper on Chloroform Anaesthesia. Dr. Richard Pearson led the discussion.

